# Successful post-term pregnancy in scared bicornuate uterus: case report

**DOI:** 10.1186/s12884-023-05875-0

**Published:** 2023-08-02

**Authors:** Tebabere Moltot, Tesfanesh Lemma, Mulualem Silesh, Moges Sisay, Birhan Tsegaw

**Affiliations:** Department of Midwifery, Asrat Woldeyes Health Science Campus, Debre Berhan University, Debre Berhan, Ethiopia

**Keywords:** Bicornuate uterus, Case report, Post-term, Uterine congenital anomalies, Cesarean section

## Abstract

Pregnancies in the bicornuate uterus are usually considered high-risk because of their association with poor reproductive outcomes such as recurrent pregnancy loss, cervical insufficiency, low birthweight, preterm birth, malpresentation, cesarean delivery, and uterine rupture. The objective of the report was to show that patients with a scared bicornuate uterus at post-term could have successful pregnancy outcomes. We are presenting a 28-year-old gravida III para II lady with a bicornuate scared uterus at post-term. She has a history of early third-trimester pregnancy loss, and her second pregnancy was delivered via cesarean section. In her first pregnancy, the patient's uterus was not identified as bicornuate. However, an ultrasound during her second pregnancy revealed that she had a bicornuate uterus, which led to the diagnosis. At term, the lady had a successful cesarean section. Lastly, for the current post-term pregnancy she had no prenatal check-up. Even though this, she was coming at the latent first stage of labor and an emergency cesarean section was done.

**Conclusion** Successful outcomes could be achieved in patients with the bicornuate uterus at post-term gestation.

## Introduction

The normal development of the reproductive organ of a female involves a series of complex processes characterized by the differentiation, migration, fusion, and subsequent canalization of the Müllerian system [1]. Abnormalities of the uterus may be congenital or acquired and typically present with menstrual dysfunction, pelvic pain, infertility, or early pregnancy loss [2]. Congenital uterine anomalies result from the abnormal formation, fusion, or resorption of Müllerian ducts during fetal life [3]. The bicornuate uterus is one of the Müllerian defects in which the fundus is indented (arbitrarily defined as ≥ 1 cm) and the vagina is generally normal [4].

A bicornuate uterus results from an incomplete fusion of the Mullerian ducts at the level of the uterine fundus, leading to two separates but communicating endometrial cavities and a single cervix. By using ultrasound bicornuate uterus is differentiated from septate uterus. The findings in the case of the septate uterus are two closely separated endometrial cavities and a smooth fundal contour (by contrast, the bicornuate uterus has an indented fundus). The depth from the interstitial line to the apex of the indentation is > 1 cm, and the angle of indentation is > 105 degrees [5].

The incidence of congenital uterine anomalies is difficult to determine since many women with such anomalies are not diagnosed, especially if they are asymptomatic [2]. The estimated prevalence of congenital uterine anomalies range from 5% in the general population [6] increasing up to more than 25% in women with recurrent miscarriage [7]. Bicornuate uterus is one of the most prevalent congenital Mullerian anomalies after arcuate uterus and uterine septum [8]. In a large review report including a combined population of infertile and fertile women, the frequency of anomalies by type was: septate (35%), bicornuate (26%), arcuate (18%), unicornuate (10%), didelphys (8%), and agenesis (3%) [9]. However, these proportions can vary substantially depending upon the specific population studied and the methodology used to identify the abnormalities [10].

Congenital uterine anomalies are strongly associated with recurrent pregnancy loss, low birth weight, preterm birth, malpresentation, cesarean delivery, and uterine rupture [11–13]. Similarly, a bicornuate uterus carries increased risks for adverse obstetrical outcomes that include miscarriage, preterm birth, malpresentation, and uterine rupture [11]. Uterine rupture in a primigravid patient with an unscarred bicornuate uterus at term is reported [10, 14]. A bicornuate uterus is an independent risk factor for cervical Os insufficiency. This is an important finding due to the burden of the risk for mid-trimester pre-viable birth associated with cervical incompetence [15]. We are not surprised only by the case of post-term pregnancy in the bicornuate uterus but also by having an intact bicornuate uterus with a previous cesarean scar at post-term (42^+5^ weeks gestational age).

## Patient information and findings

A 28-year-old gravida III Para II (one alive, one stillbirth) illiterate housewife with a diagnosis of the latent first stage of labor, a previous caesarean scar, and breech presentation was referred from Fetra Health Center. In 2016, she gave birth to a female fetus weighing 1.1 kg at a gestational age (GA) of 28 weeks from the last normal menstrual period at Fetra health center. In 2019 she was coming with the second pregnancy at Fetra health center at the latent first stage of labor and was referred to Enat hospital with an assessment of transverse lie. She was found to have a bicornuate unicollis uterus at Enat Hospital by ultrasonography with transverse lying, and the fetus was found to be in the right horn of a bicornuate uterus. Emergency cesarean section was done to effect a 2.5 kg male alive neonate with an APGAR score of 6 and 8 in the first and fifth minutes. She had a smooth post-operative recovery and was discharged on the fourth day of the operation with a brief description of the procedure and strong advice about the next pregnancy, place and mode of delivery.

By now, (in 2022), she was referred with the above diagnosis (Latent first stage of labor + Previous cesarean scar + Breech presentation) from the health center. During her pregnancy, she had no antenatal care (ANC) contact. Her first day of regular last normal menstrual period (LNMP) was on September 11/2021 making her GA = 42^+5^ weeks. Ultrasonography revealed that the fetus was found to be in the right horn of a bicornuate uterus with a transverse lie (shoulder presentation). It also revealed that an amniotic fluid index of 6 cm, and grade three placental calcification. The depth of the indentation is 4 cm, and the angle of indentation is around 120 degrees.

**Fig. 1:**
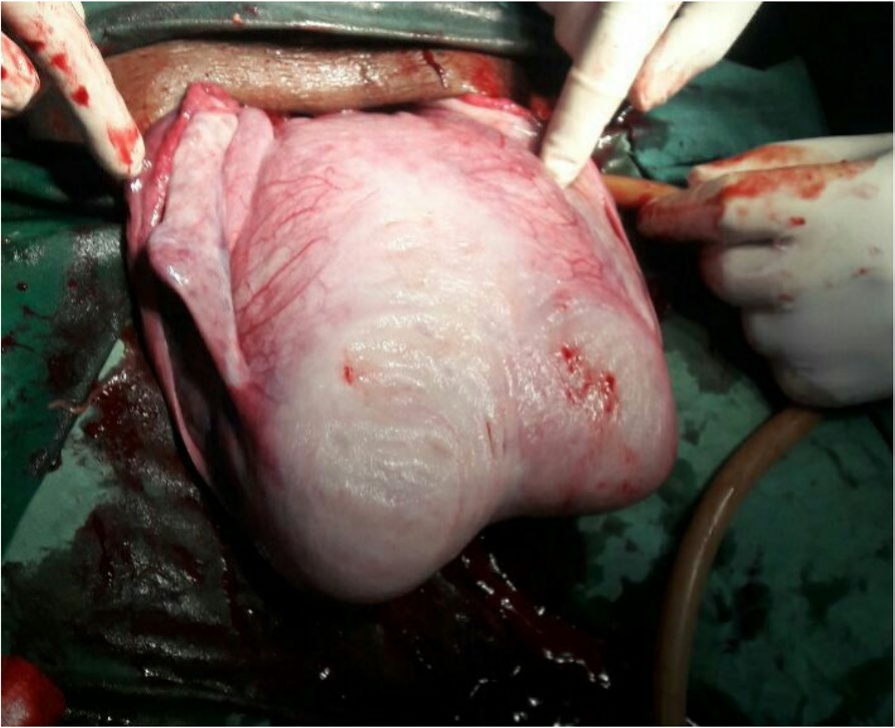
The right horn that contained the baby is obviously bigger than the left, posterior wall, 2022

**Fig. 2:**
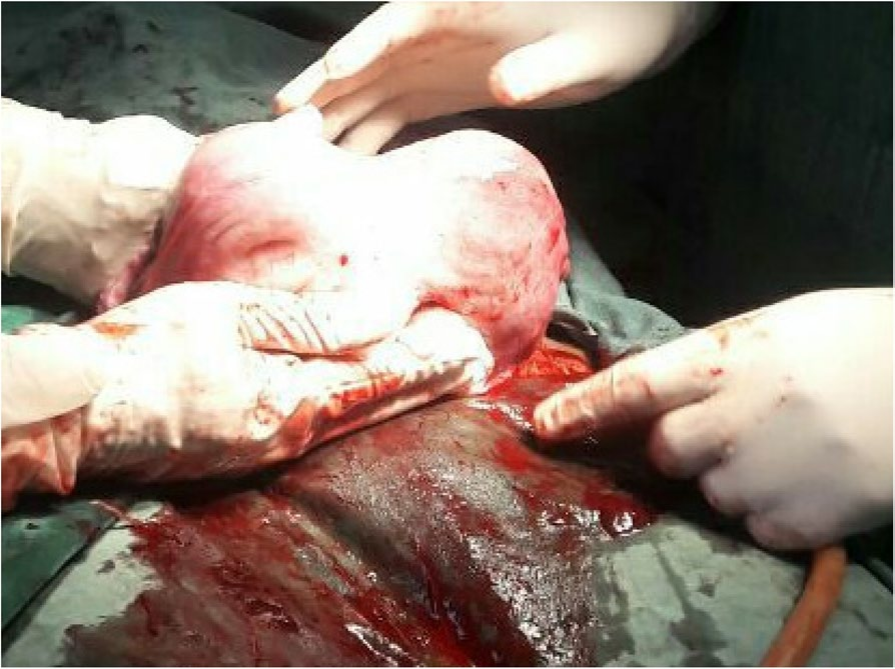
The exteriorized bicornuate uterus showing the cesarean incision on the bicornuate uterus after the delivery of the baby, 2022

Following informed written consent and spinal anesthesia, an emergency cesarean section was performed to effect a 2.6 kg male alive neonate with an APGAR score of 7 and 9 in the first and fifth minutes, respectively. The cesarean section was done for an indication of latent first stage of labor + transverse lie + bicornuate unicollis scared uterus + post-term. Intraoperative findings: a fetus in the right horn, grade II meconium, and an intact gravid bicornual uterus with a well-formed lower uterine segment. The placenta was anterior and fundal, and both uterine tubes and the ovaries were grossly normal. The estimated blood loss during surgery was 650 ml (Figs. 1 and 2).

The neonate exhibited post-maturity (i.e., dry, loose, and peeling skin, overgrown nails, minimal fat deposit, and wide-eyed). Both the neonate and the mother were discharged home on the fourth day, with an appointment for the 7th post-operative day. The postnatal follow‑up revealed that no abnormality on both the mother and neonate. The mother was counselled on family planning methods, issues related to the next pregnancy; early, and close ANC follow-up. She also counselled about place of delivery and possibility of early uterine rupture in the next pregnancy. Lastly Implanon was provided by her choice and she was discharged from the postnatal clinic.

She has no personal and family history of chronic illness like hypertension, diabetics, renal and cardiac disease.

Result of lab investigation revealed that her Blood group and Rh was A^+^, her HIV, Hepatitis B virus, and syphilis result is non-reactive and all CBC result was in normal range.

## Discussion

Congenital uterine anomalies are strongly associated with recurrent pregnancy loss, low birth weight, preterm birth, malpresentation, cesarean delivery, and uterine rupture [11–13]. The bicornuate uterus was previously thought to be associated with infertility [16]. However, as recent studies showed, fertility in women with a bicornuate uterus is unaffected, but gestational capacity is impaired because of the high number of spontaneous abortions, and preterm deliveries [17, 18]. Our case also had a previous early third-trimester pregnancy loss (at GA of 28 weeks). Since viable birth rates progressively increase in young women with a bicornuate uterus and no other factor compromising fertility. After one or two abortions metroplasty is questionable, rather cervical cerclage is attempted to avoid second-trimester abortion or premature delivery [17]. It has been reported that women with a bicornuate uterus and repeated pregnancy losses may benefit more from metroplasty than from cerclage [19]. At subsequent pregnancy, the case was coming to term with transverse lie and ultrasound revealed the fetus to be in the right horn of a bicornuate uterus. Emergency cesarean section was done to effect the normal fetus. The inadequate room for rotation can be the explanation for the fetal malpresentation that is associated with bicornuate uterus [20]. Studies revealed that having of neonatal limb deformity after giving a viable fetus [1, 21]. The fetal limb deformity might result from prolonged pressure on the limbs due to a lack of space within the uterine horn where fetal development took place.

Her third pregnancy was in the right horn of a bicornuate uterus with a transverse lie. Under spinal anesthesia emergency cesarean section was done for an indication of transverse lie + bicornuate unicollis scared uterus + post-term, to effect male alive neonate with a feature of post maturity. As in the case presented, several cases of successful pregnancies in the bicornuate uterus had been reported without surgical correction of the anomaly [16, 20]. But as far as our review there was no report of post-term pregnancy with scared bicornuate uterus. Regarding to other complications, uterine rupture in a primigravid patient with an unscarred bicornuate uterus at term is reported. A rupture of the uterus ends up with a cesarean hysterectomy and loss of fertility [10, 14]. In the other case, in term (even preterm) bicornuate pregnant uterus fetal limb deformity might result from prolonged pressure on the limbs due to lack of space within the uterine horn [20]. But in the above-presented case, the bi-cornuate scared gravid uterus was intact with a well-formed lower uterine segment which make the woman lucky.

Transvaginal 3-D ultrasonography (US) appears to be extremely accurate for the diagnosis and classification of congenital uterine anomalies, more than office hysteroscopy and MRI [22]. Detailed examination of the uterine septum using experienced hands, by 3-D US has a high reproducibility for diagnosing uterine anomalies (specially septate and bicornuate uterus) for preoperative planning [23]. In contrast, bicornuate and septate uterine anomalies are less confidently differentiated *by* traditional 2-D TVS techniques. Ideally, the angle between the two endometrial cavities is ~ 105°for the bicornuate uterus, but 75° for the septate uterus. The fundal shape shows a > 1 cm notch for the bicornuate uterus, but a < l cm notch for the septate uterus [24]. In our case, the finding showed that the depth of the indentation is 4 cm, and the angle of indentation is around 120 degrees. Which is confirmed by actual measurement of indentation using calibrated forceps intraoperatively.

## Conclusion

A successful outcome could be achieved in patients with the bicornuate uterus at post-term gestation and if no other factor compromises fertility**,** the viable birth rate progressively increases for women with a bicornuate uterus.

## Limitation

Initially, we didn’t consider the case for the case report, and we lost supporting images of ultrasound.

## Patient perspective

I am a 28-year-old illiterate housewife. I knew since I have an abnormal uterus three years back during the time of second delivery by operation. At the time of discharge, the physician told me to took family planning and have an ANC follow-up when I am pregnant. I delayed starting ANC follow up for this pregnancy because I feeling fine and the health facility is so distant from my village. However, I was headed to a medical institution when labor started, the second procedure had already been completed. I learned a valuable lesson from this, and moving forward, I'll follow your advice for the next.

## Data Availability

All relevant data are within the manuscript and its supporting information files.

## Abbreviations

ANC: Antenatal care
GA: Gestational age
US: Ultra sonography
